# Next‐Generation Drug Delivery Systems for Alzheimer's Disease: Overcoming Barriers to Effective Treatment

**DOI:** 10.1002/alz70860_100746

**Published:** 2025-12-23

**Authors:** Sukriti Vishwas, Sachin Kumar Singh, Bushra Bashir

**Affiliations:** ^1^ Lovely Professional University, Jalandhar, India; ^2^ School of Pharmaceutical Sciences, Lovely Professional University, Jalandhar, Punjab, India

## Abstract

**Background:**

Alzheimer's disease (AD) is a neurodegenerative disorder characterized by cognitive dysfunction. Key contributing factors include amyloid‐beta plaques, tau tangles, and neuroinflammation. Although multiple drugs have been tested in preclinical and clinical studies, their effectiveness is often limited by poor dissolution in the gut and low permeability across the blood‐brain barrier (BBB). Next‐generation drug delivery systems have been developed to improve targeting, bioavailability, brain permeability, and therapeutic responses.

**Method:**

The above study highlights advanced drug delivery systems (ADDS) for treating AD. Various systems, including polymeric, lipid, and inorganic nanoparticles, were examined for their ability to cross the BBB. Functionalized nanoemulsions with ligands such as transferrin were evaluated for enhanced targeting, while intranasal delivery methods were assessed for their potential to facilitate direct access to the brain. Innovative approaches, including exosome‐based systems, hydrogels, microneedles, and stimuli‐responsive platforms, were analyzed for their roles in controlled and sustained drug release. Furthermore, gene‐based therapies like miRNA, siRNA, CRISPR/Cas9, and monoclonal antibodies, were explored for their effectiveness in targeting amyloid beta and neurofibrillary tangles.

**Result:**

In preclinical and clinical studies ADDS deciphered neuroprotective effects for the treatment of AD with improved drug bioavailability, reduced amyloid aggregation, enhanced cognitive function, etc.

**Conclusion:**

Next‐generation drug delivery systems presents innovative solutions for addressing the challenges associated with the treatment of AD. They pave the way for more effective and targeted therapeutic interventions.

